# Treatment patterns and outcomes in perineal primary vulvar cancer—a population‐based Swedish cohort study

**DOI:** 10.1111/aogs.70315

**Published:** 2026-07-22

**Authors:** Karin Kjölhede, Gabriel Lindahl, Christian Staf, Diana Zach, Katja Stenström Bohlin

**Affiliations:** ^1^ Department of Obstetrics and Gynecology Sahlgrenska University Hospital Gothenburg Sweden; ^2^ Department of Oncology, and Department of Biomedical and Clinical Sciences Linköping University Linköping Sweden; ^3^ Regional Cancer Center Gothenburg Sweden; ^4^ Department of Gynecological Cancer Karolinska University Hospital Stockholm Sweden; ^5^ Department of Women's and Children's Health Karolinska Institutet Stockholm Sweden; ^6^ Department of Obstetrics and Gynecology, Institute of Clinical Sciences Sahlgrenska Academy, University of Gothenburg Gothenburg Sweden

**Keywords:** complications, perineum, radiotherapy, recurrence, survival, vulvar cancer

## Abstract

**Introduction:**

The choice of treatment for vulvar squamous cell carcinoma involving the perineum is challenging because of its anatomical proximity to the anus. The study aim was to describe treatment patterns, complications, and survival of women with perineal vulvar squamous cell carcinoma.

**Material and Methods:**

A population‐based study at two tertiary referral hospitals during 2012–2020. Demographic and clinical data were retrieved from medical records. Survival and complications were analyzed according to treatment modality. Relative and progression‐free survival were calculated by Kaplan–Meier estimates.

**Results:**

The cohort comprised 571 women with primary vulvar squamous cell carcinoma, of which 147 (26%) were located at the perineum. Seventy‐eight women (78/147; 53%) underwent surgery only with a median resection margin of 5 mm. In 34/147 women (23%), surgery was followed by adjuvant radiotherapy because of lymph node metastasis (74%) and/or insufficient margins (median resection margin 1.6 mm). Primary radiotherapy was delivered to 35/147 women (24%). Treatment‐related complications during the first year were recorded in 47% after surgery only, in 79% after adjuvant radiotherapy and in 71% after primary radiotherapy. After a median follow‐up of 41 months, disease progression occurred in 3% of women treated with surgery only, in 18% after adjuvant radiotherapy and in 33% after primary radiotherapy. The 2‐year progression‐free survival was 82% (95% CI, 74–91) after surgery only, 53% (95% CI, 38–73) after adjuvant radiotherapy, and 31% (95% CI, 19–51) after primary radiotherapy.

**Conclusions:**

When diagnosed at an early‐stage, perineal vulvar squamous cell carcinoma can successfully be treated by primary surgery. The optimal treatment strategy for advanced disease remains uncertain and requires further investigation.

## Graphical abstract

Perineal vulvar squamous cell carcinoma was identified in 147 of 571 cases. Owing to the anatomical proximity of these tumors to the anus, treatment selection may present considerable clinical challenges. The majority of patients (53%) underwent surgical treatment alone, whereas 23% received surgery combined with adjuvant radiotherapy and 24% were treated with primary radiotherapy.

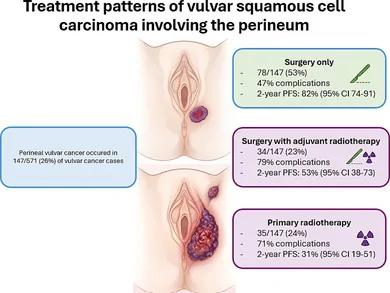

**Abbreviations**
RTOG: Radiation Therapy Oncology Group
SQRGC: Swedish Quality Registry of Gynecologic Cancer

**Key message**

Treatment of perineal vulvar squamous cell carcinoma is challenging and accompanied by high complication rates. Early‐stage disease can often be successfully managed with surgery. Optimal treatment strategy for advanced disease requires further investigation.

## 1 INTRODUCTION

Vulvar squamous cell carcinoma is a relatively rare disease with an estimated global incidence of 47 000 women in 2022.^1^ It accounts for 5% of all gynecological cancers and predominantly affects older women. The incidence is increasing, largely due to human papillomavirus (HPV)‐associated disease in younger women.^2^ The overall 5‐year survival rate is approximately 70%, with lymph node metastasis being the most important prognostic factor.^3^ The anatomical location of the tumor may also influence prognosis. Clitoral and urethral involvement appear to have a negative impact on survival, whereas primary perineal tumors may be associated with a more favorable prognosis due to differences in histopathological characteristics and lymph node metastatic patterns.^4^, ^5^ The definition of a perineal tumor is heterogeneous or absent across studies, contributing to variation in the reported prevalence of 5–18%. Some studies classify perineal tumors solely based on the tumor epicenter being located in the posterior commissure,^6^ whereas others include all vulvar tumors involving the perineal region.^4^, ^7^, ^8^

In general, the preferred treatment for vulvar squamous cell carcinoma is wide local excision with inguinal lymph node assessment.^9^, ^10^ However, when the perineum is involved, excision often must be performed close to the external anal sphincter, increasing the risk of inadequate surgical margins and the subsequent need for adjuvant radio(chemo)therapy to the vulva. Alternatively, achieving adequate margins may require more radical surgery, including resection of the anus and fecal deviation with a permanent stoma. Primary radio (chemo)therapy represents an alternative to extensive surgery.^11^ Consequently, selecting a treatment that provides oncological safety while preserving anal sphincter function can be challenging. To date, there is no consensus on the optimal management of perineal vulvar squamous cell carcinoma, and population‐based data on treatment patterns for tumors at this specific location are scarce.

The purpose of this study was to describe the treatment pattern of women with vulvar squamous cell carcinoma involving the perineum in a population‐based cohort with respect to treatment‐related complications, recurrence, and survival.

## 2 MATERIAL AND METHODS

### 2.1 Study population

This population‐based observational study included all women with primary vulvar squamous cell carcinoma involving the perineum who were treated at Sahlgrenska University Hospital or Linköping University Hospital between January 1, 2012 and December 31, 2020. All stages and treatment modalities were included. Patients who did not receive treatment or whose treatment modality could not be determined were excluded. The hospitals serve a catchment area of approximately four million inhabitants and, following the centralization of vulvar cancer care in 2017, represent two of four vulvar cancer centers in Sweden. In Sweden, healthcare is tax‐funded, and all women with vulvar cancer are treated at publicly funded university hospitals.

### 2.2 Data

The women were identified through the Swedish Quality Registry of Gynecologic Cancer (SQRGC), a national quality registry linked to the mandatory National Swedish Cancer Registry, which covers 97%–98% of malignant gynecological cancers in Sweden.^12^ Registration of vulvar cancer in the SQRGC started in 2012. Patient consent for inclusion in the SQRGC is presumed and no written consent is required. Written information is provided at admission, and patients may request withdrawal from the registry at any time.

The medical records of the women were reviewed. Perineal involvement was defined as any tumor involving the perineal area, including tumors extending onto the labia, provided that the predominant tumor growth was located in the perineum. Tumors located in the upper vulvar region or the labia without perineal involvement were excluded. The following variables were collected: age at diagnosis, smoking status, body mass index (BMI), history of lichen sclerosus, tumor size, International Federation of Gynecology and Obstetrics (FIGO 2009) stage,^13^ and primary treatment modality. In addition, treatment‐related complications, follow‐up duration, and recurrence data were collected. Pathology reports were reviewed for information on histopathological tumor type, tumor differentiation grade, tumor size, and depth of invasion, surgical margins, p16 immunohistochemical staining status, coexisting lichen sclerosus, and the presence of lymph node metastasis. The surgical margin was defined as the shortest histopathologically tumor‐free margin in the final specimen.

Treatment was administered according to the regional and national Swedish guidelines for vulvar cancer that were in force at the time and were aligned with the recommendations of the European Society of Gynecological Oncology. Cases were discussed either at a regional multidisciplinary team (MDT) conference or, following centralization of care in 2017, at a national MDT conference.

Radiotherapy (RT) was delivered using three‐dimensional conformal RT (3D‐RT), intensity‐modulated RT (IMRT), or volumetric‐modulated arc therapy (VMAT), according to local practice. Recommended follow‐up consisted of visits every 6 months during the first 3 years after treatment and annually thereafter for an additional 2 years. Both study centers offered outpatient appointments within a cancer rehabilitation program.

Complications were categorized according to the time from primary treatment: within 30 days, 1–6 months, and 7–12 months. Complications were included if they were considered clinically relevant, defined as persistent complications, or complications requiring treatment, or complications of Clavien Dindo grade > 2.^14^ Radiation dermatitis was graded according to the Radiation Therapy Oncology Group (RTOG) scale, and grade ≥ 3 dermatitis was considered a complication.^15^

Time to recurrence was defined as the interval between treatment initiation and the diagnosis of recurrence. Patients who had not achieved disease‐free status within 6 months of treatment completion were excluded from this analysis.

### 2.3 Statistics

The central tendency and dispersion of continuous data were presented as median and range. Nominal data were reported as numbers and percentages. Continuous variables were compared using non‐parametric tests, namely the Mann–Whitney *U*‐test or Kruskal–Wallis analysis of variance, as appropriate. Pearson's chi‐square test was used to compare categorical variables between groups. A two‐sided *p*‐value of < 0.05 was considered statistically significant. No imputation methods were applied.

Kaplan–Meier curves were used to estimate relative survival and progression‐free survival. Mortality data from the general Swedish population were used to calculate expected survival rates for the study population. Relative survival was defined as the time from diagnosis to death or, for patients who were still alive, to the date of the last data collection (April 2022). Information on date of death is continuously updated in the SQRGC through linkage with the National Cause of Death Register. Progression‐free survival was defined as the time from initiation of primary treatment to the first recurrence, progression, death, or censored at last follow‐up. Statistical analyses were performed using IBM SPSS Statistics for Windows, version 24.0 (IBM Corporation, Armonk, NY), and R statistical software, version 4.33. The R package *survival* (version 3.5.8) and the package *relsurv* (version 2.29) were used to estimate relative and progression‐free survival respectively.

## 3 RESULTS

A total of 571 women with vulvar cancer were identified, of whom 160 (28%) had vulvar squamous cell carcinoma with perineal involvement. After excluding patients who received best supportive care, the final study cohort comprised 147 of 571 women (26%) who underwent primary treatment. The complete cohort of women with primary vulvar cancer treated at the two participating hospitals, as well as the selection of the study cohort and treatment modalities are depicted in the flow chart (Figure 1).

**FIGURE 1:**
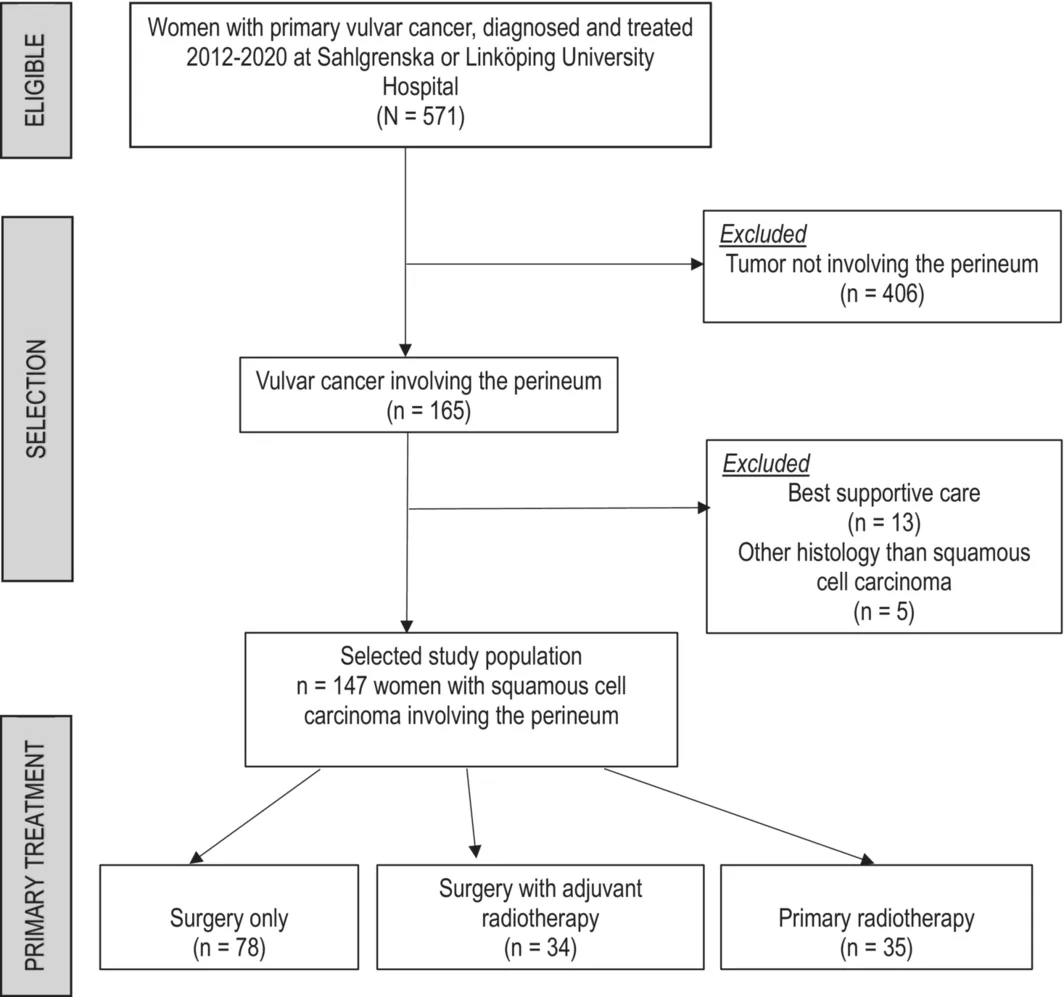
Flow chart of the study population.

Demographic, clinical, tumor, and treatment characteristics are presented in Table 1. The women were grouped according to primary treatment: (1) vulvar surgery only with or without inguinofemoral lymph node assessment (*n* = 78/147, 53%), (2) surgery followed by adjuvant radiotherapy (*n* = 34/147, 23%), and (3) primary radiotherapy (*n* = 35/147, 24%).

**TABLE 1:** Demographic and clinical characteristics of women with perineal vulvar squamous cell carcinoma in relation to treatment modality.

Treatment	Surgery only *n* = 78	Surgery + adjuvant radiotherapy *n* = 34	Primary radiotherapy *n* = 35	*p*‐value
Age, years	68.5 [25.0–95.0]	74.5 [44.0–90.0]	74.0 [41.0–92.0]	0.12^a^
BMI, kg/m^2^	25.6 [17.0–44.0]	26.6 [19.9–43.1]	26.5 [16.0–40.0]	0.26^a^
Smoking	22 (28.2)	8 (23.5)	7 (20.0)	0.32^b^
FIGO stage				
IA	18 (23.1)	0	0	< 0.01^b^
IB	51 (66.2)	3 (8.8)	1 (2.9)	
II	4 (5.2)	5 (14.7)	7 (20.0)	
III	5 (6.4)	24 (67.6)	14 (40.0)	
IV	0 (0.0)	3 (8.8)	13 (37.1)	
Clinical vulvar tumor size, cm	3.0 [0.4–12.0]	4.0 [2.0–9.0]	5.8 [2.0–16.0]	< 0.01^a^
< 4 cm	44 (56.4)	10 (29.4)	3 (8.6)	< 0.01^b^
≥ 4 cm	26 (33.3)	24 (70.6)	31 (88.6)	
Missing data	8 (10.4)	0 (0.0)	1 (2.9)	
Flap repair	0 (0.0)	3 (8.8)	NA	
Posterior pelvic exenteration	3 (3.8)	1 (2.9)	NA	
Defunctioning stoma	NA	NA	3 (8.6)	
Groin surgery				
Yes	53 (69.2)	31 (91.2)	NA	
IFL	32 (59.3)	26 (83.9)	NA	
SLN only	22 (40.1)	5 (16.1)	NA	
No	24 (30.8)	3 (8.8)	NA	
Pathology report				
Depth of invasion mm	3.20 [0.1–31.0]	9.65 [1.6–32.0]	NA	< 0.01^c^
Surgical margins^d^, mm	5.00 [0.0–15.0]	1.60 [0.0–9.0]	NA	< 0.01^c^
p16 staining (any biopsy)				
Positive	17 (22.1)	3 (8.6)	6 (17.1)	< 0.01^b^
Negative	3 (3.9)	4 (11.4)	2 (5.7)	
Missing data	57 (74.0)	28 (80.0)	27 (77.1)	
Lichen sclerous^e^				
Yes	20 (26.0)	10 (29.4)	6 (17.1)	< 0.01^b^
No	58 (74.4)	23 (67.6)	23 (65.7)	
Missing data	0	1 (2.9)	6 (17.1)	
Radiotherapy only	NA	20 (57.1)	17 (48.6)	
Chemoradiotherapy	NA	15 (44.1)	18 (51.4)	
Radiation treatment field				
Vulva	NA	5 (21.1)	12 (34.3)	
Groins	NA	4 (15.8)	0	
Both vulva and groins	NA	25 (63.2)	23 (65.7)	

*Note*: Figures denote median and [range] or number and (percent).

Abbreviations: BMI, Body mass index; FIGO, International Federation of Gynecology and Obstetrics 2009; IFL, inguinofemoral lymphadenectomy; NA, not applicable; SLN, sentinel lymph node dissection.

^a^ Kruskal‐Wallis test.

^b^ Pearson's chi‐square test.

^c^ Mann–Whitney U‐test.

^d^ In the surgical specimen.

^e^ In medical history or in surgical specimen.

The treatment groups did not differ significantly with respect to age, BMI, or smoking status but differed in stage distribution. The distribution of FIGO (2009) stage was as follows: stage IA, 12%; stage IB, 37%; stage II, 11%; stage III, 29%; and stage IV, 11%. Nearly 90% of patients in the surgery only group had FIGO (2009) stage I disease, whereas more than 75% of patients in the other two groups had advanced‐stage disease (*p <* 0.01) or tumors larger than 4 cm (*p <* 0.01). Groin staging surgery was performed in 76% of women who underwent surgery and was mainly omitted because of stage IA disease or medical comorbidities. Seven women (4.8%) underwent colostomy or enterostomy; three received a defunctioning stoma prior to primary radiotherapy, and four following modified posterior pelvic exenteration.

Adjuvant radiotherapy was administered to 30% of women who underwent primary surgery. The median free surgical margin was narrower in the surgery + adjuvant radiotherapy group compared to the surgery only group (1.6 mm vs. 5 mm, *p* < 0.01). In the adjuvant radiotherapy group, 74% had lymph node metastases, and in all but 4 cases, the vulva was included in the radiation field. Radiotherapy was delivered exclusively to the vulva in five women because of inadequate surgical margins (range 0–2.5 mm). Although recommended by treatment guidelines, five women in the surgery only group with FIGO stage III disease did not receive adjuvant radiotherapy because of patient‐related factors. The median radiation dose delivered to women receiving adjuvant radiotherapy was 50 Gy (34.0–66.0 Gy). Women who underwent primary radiotherapy with curative intent (*n* = 27, 77%) received a median dose of 66 Gy (45.0–72.0 Gy), whereas those treated with palliative intent (*n* = 8, 23%) received a median dose of 42.0 Gy (16.0–50.0 Gy). Nearly half of the women undergoing radiotherapy (48%) were treated with concomitant chemotherapy.

There was a significant difference in the prevalence of HPV‐related vulvar squamous cell carcinoma among the three treatment groups (*p <* 0.01) with a lower prevalence observed in the adjuvant radiotherapy group. However, less than 30% of the cohort had been analyzed for p16 status. The prevalence of lichen sclerosus, diagnosed clinically or histopathologically, was 25% and was higher in the surgery only and adjuvant radiotherapy groups than in the primary radiotherapy group (*p <* 0.01).

Complications were more frequent among women receiving radiotherapy than among those treated with surgery only (*p* < 0.01) (Table 2). Wound infection, particularly involving the vulva, was the most frequent postoperative complication. Severe radiation dermatitis was a common complication after radiotherapy. There was no significant difference in the occurrence of lymphedema between the groups. Fecal incontinence was rarely reported in the medical records. Only three women visited oncological rehabilitation services after primary treatment; one after primary surgery only and two after adjuvant radiotherapy.

**TABLE 2:** Complications according to modality of treatment in women with perineal vulvar squamous cell carcinoma.

Treatment	Surgery only *n* = 78	Surgery + adjuvant radiotherapy *n* = 34	Primary radiotherapy *n* = 35	*p*‐value^a^
Number of women with any complication within first year of treatment	37 (47.4)	27 (79.4)	25 (71.4)	< 0.01
Within 30 days	25 (32.1)	14 (41.2)	22 (62.9)	< 0.01
Between 1 and 6 months	10 (12.8)	20 (58.8)	4 (11.4)	< 0.01
Between 7 and 12 months	5 (6.4)	9 (26.5)	2 (5.7)	< 0.01
Type pf complication				
Any vulvar wound complication	21 (26.9)	11 (32.4)	5 (14.3)	0.39
Wound infection	18 (23.1)	8 (23.5)	5 (14.3)	
Dehiscence	9 (11.5)	7 (20.6)	NA	
Any groin wound complication	11 (14.1)	10 (29.4)	2 (5.7)	0.08
Wound infection	11 (14.1)	9 (26.5)	2 (5.7)	
Dehiscence	3 (3.8)	3 (8.8)	NA	
Infection of unknown location	1 (1.3)	0	1 (2.9)	
Radiation dermatitis RTOG ≥ 3	NA	14 (41.2)	22 (62.9)	0.01
Fecal incontinence	1 (1.3)	3 (8.8)	1 (2.9)	
Lymphedema	8 (10.3)	6 (17.6)	4 (11.4)	0.75
Vulva	3 (3.8)	1 (2.9)	1 (2.9)	
Lower extremities	2 (2.6)	3 (8.8)	1 (2.9)	
Both vulva and lower extremities	3 (3.8)	2 (5.9)	2 (5.7)	
Necrotizing fascitis	0 (0.0)	1 (1.3)	0 (0)	
Intestinal perforation^b^	0 (0.0)	0 (0.0)	1 (2.9)	
Fistula^c^	0 (0.0)	2 (5.9)	0 (0.0)	
Ileus	0 (0.0)	0 (0.0)	1 (2.9)	

*Note*: Figures denote number and (percent).

Abbreviations: NA, not applicable; RTOG (radiation therapy oncology group) scale.

^a^ Pearson's chi‐square test.

^b^ After insertion of suprapubic catheter.

^c^ One anovaginal and one rectovaginal fistula.

### 3.1 Survival and recurrence

The median follow‐up time was 41 months (range, 0–96 months). Data on recurrence are presented in Table 3. Within the first 6 months after treatment, 3% of women treated with surgery only and 18% of those treated with adjuvant radiotherapy experienced disease progression. Among the 35 women who received primary radiotherapy, eight were treated with palliative intent, and 75% of these women had residual tumor following treatment. Among women who received primary radiotherapy with curative intent, 33% experienced disease progression within 6 months.

**TABLE 3:** Recurrence data in perineal vulvar squamous cell carcinoma.

	Surgery only	Surgery + adjuvant radiotherapy	Primary radiotherapy	*p*‐value
	*n* = 78	*n* = 34	*n* = 35	
NED 6 months after primary treatment				
No	2 (2.6)	6 (17.6)	15 (42.9)	<0.01^a^
Yes	76 (97.4)	28 (82.4)	20 (57.1)	
Recurrence among women with NED	6/75 (7.9)	12/28 (42.9)	9/20 (45.0)	<0.01^a^
Months to recurrence [range]^c^	18.0 [11–72]	11.5 [6–62]	11.0 [7–53]	0.66^b^
Months from recurrence to death [range]	4.0 [3–83]	5.0 [2–66]	7.0 [2–33]	0.78^b^
Site of first recurrence				
Isolated vulvar	4/6 (66.7)	7/12 (58.3)	6/9 (66.7)	
Recurrence within previously radiated field	NA	7/7 (100)	6/6 (100)	
Isolated groins	0 (0.0)	3/12 (25.0)	2/9 (22.2)	
Recurrence within previously radiated field	NA	3/3 (100)	1/2 (50)	
Vulvar + groins	1/6 (16.7)	1/12 (8.3)	0 (0.0)	
Recurrence within previously radiated field	NA	1/1 (100)	0 (0.0)	
Distant	1/6 (16.7)	1/12 (8.3)	1/9 (11.1)	

*Note*: Figures denote median and [range], or number and (percent).

Abbreviations: NED, no evidence of disease 6 months after completed primary treatment; NA, not applicable.

^a^ Pearson's chi‐square test.

^b^ Kruskal‐Wallis test.

^c^ From start of primary treatment.

Of the 124 women who achieved complete remission, 22% experienced a recurrence, with most recurrences occurring in the radiotherapy groups. There was one groin recurrence in the surgery only group and one patient with distant metastasis; the latter had declined adjuvant radiotherapy. At the time of data collection, 82% of the women with recurrent disease had died.

The total cohort had a 2‐year relative survival of 71% (95% CI, 63–79). The corresponding 2‐year relative survival rates were 87% (95% CI, 79–96) in the surgery only group, 64% (95% CI, 49–83) in the adjuvant radiotherapy group, and 42% (95% CI, 28–63) in the primary radiotherapy group (Figure 2). The 2‐year progression‐free survival was 82% (95% CI, 74–91) in the surgery only group, 53% (95% CI, 38–73) in the adjuvant radiotherapy group, and 31% (95% CI, 19–51) in the primary radiotherapy group (Figure 3). After excluding women treated with palliative intent in the primary radiotherapy group, the 2‐year relative survival was 46% (95% CI, 30–69) (Figure S1) and the 2‐year progression‐free survival was 37% (95% CI, 23–61) (Figure S2).

**FIGURE 2:**
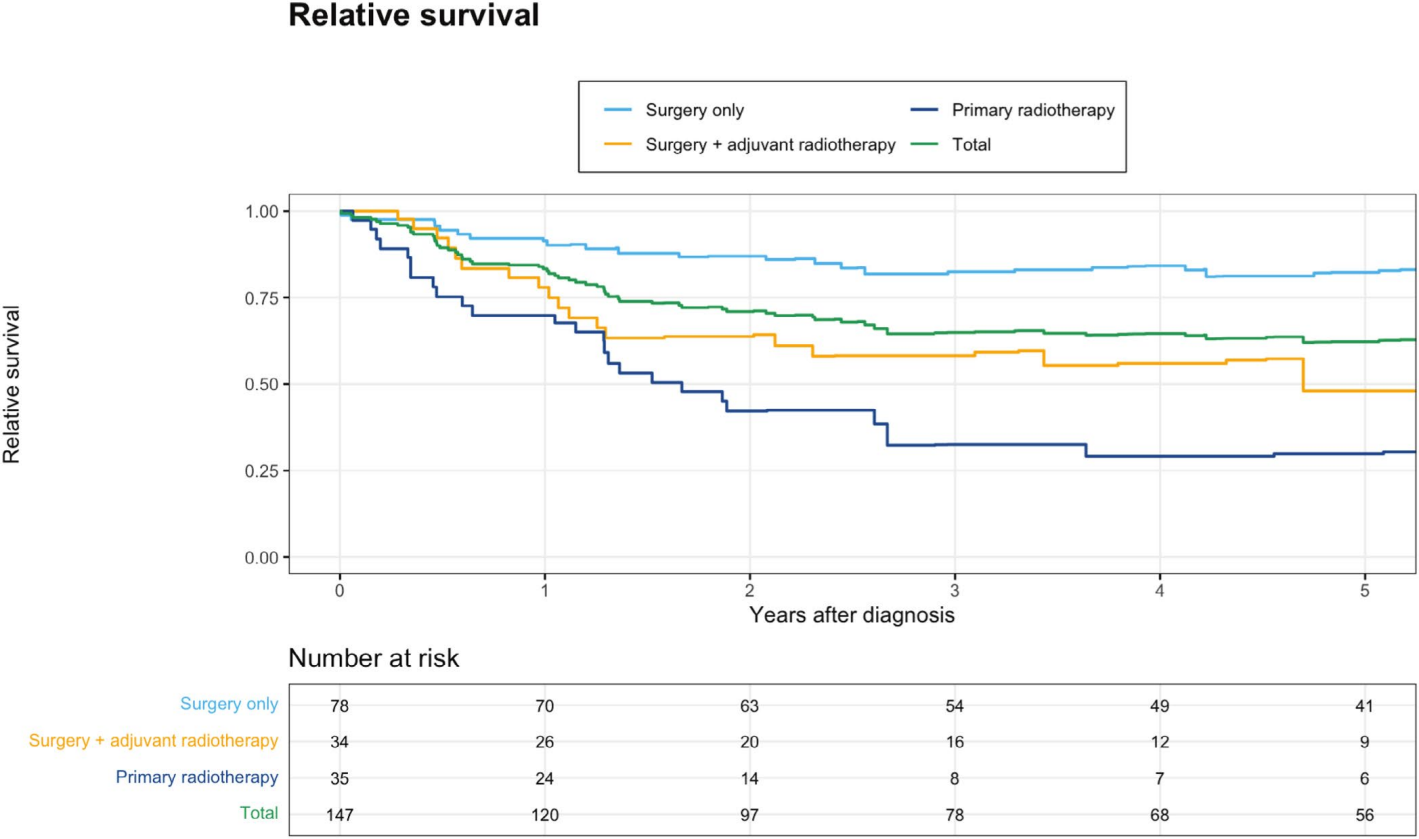
Relative survival among patients according to treatment modality of perineal vulvar cancer.

**FIGURE 3:**
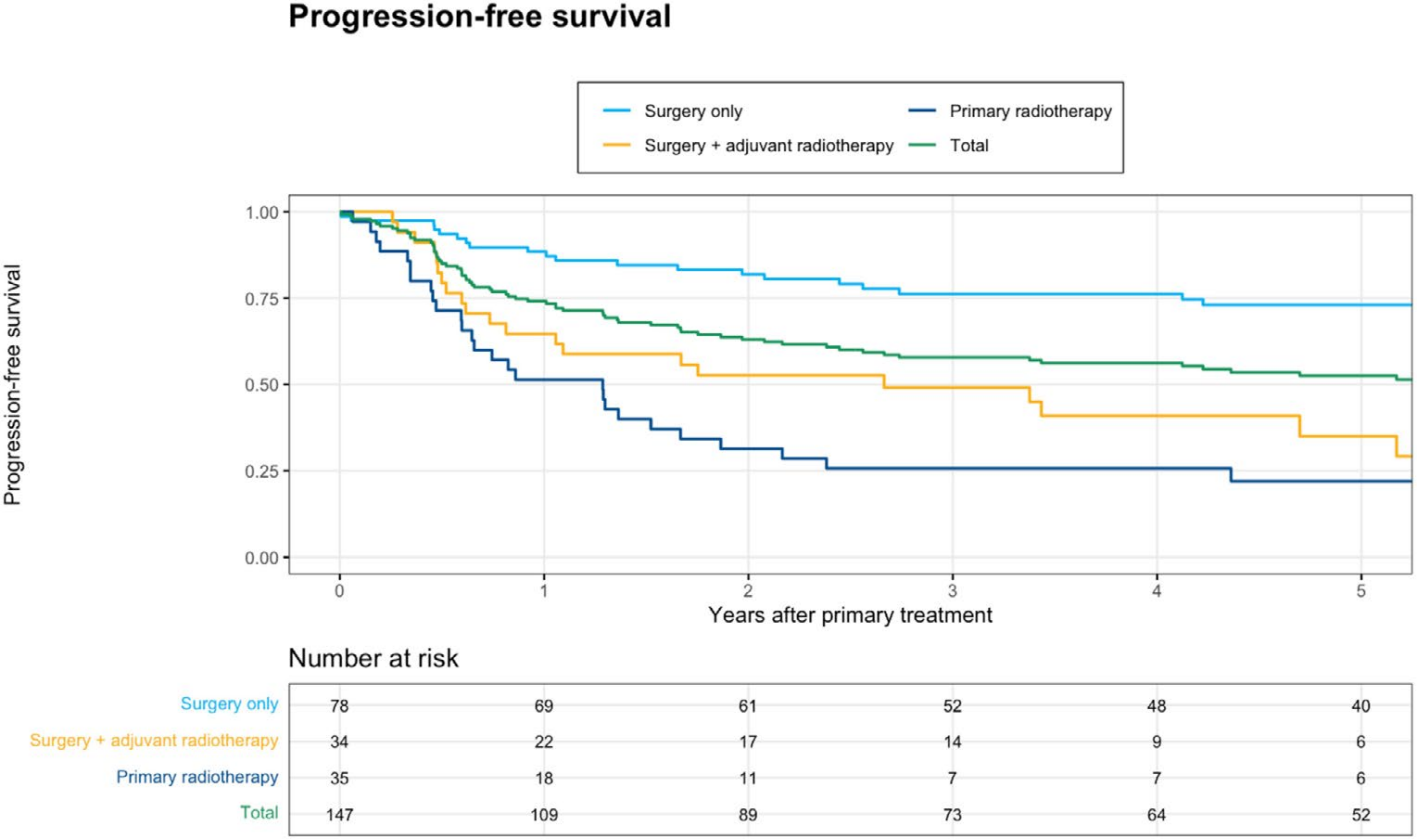
Progression‐free survival according to treatment modality of perineal vulvar cancer.

## 4 DISCUSSION

This population‐based study describes treatment patterns and outcomes among women with vulvar squamous cell carcinoma involving the perineum. About a fourth of women with newly diagnosed vulvar cancer had tumors involving the perineum. The treatment groups differed significantly in stage distribution, and treatment patterns reflected disease severity. Although most women underwent surgical treatment, a substantial portion received either adjuvant or primary radiotherapy. Stomas and posterior modified exenterations were performed in fewer than 5% of cases. Adjuvant radiotherapy was administered primarily because of lymph node metastases, whereas narrow surgical margins were rarely the sole indication. Primary radiotherapy was mainly delivered to women with advanced‐stage disease and large tumors. Relative and progression‐free survival mirrored the stage distribution, with favorable outcomes in the surgery only group, which predominantly comprised women with early‐stage disease and poorer outcomes among women requiring radiotherapy for more advanced disease.

Previous studies including perineal vulvar cancer were conducted at single centers and limited to surgically treated patients.^4^, ^6^, ^7^, ^8^ In the present study, any tumor involving the perineum was considered, regardless of extension to the labia. The rationale was to include tumors in close proximity to the anal region that could pose specific challenges in treatment planning. Furthermore, all treatment modalities, including radiotherapy, were evaluated, which may explain the higher prevalence of perineal tumors (26%) observed compared with previous studies (5–18%). In addition, the population‐based design ensured inclusion of a complete regional cohort.

A previous Swedish study by Hellman et al. examining primary treatment patterns for vulvar cancer across all anatomical locations during 2012–2016 reported that surgery only was performed in 60% of patients, 21% underwent surgery followed by adjuvant radiotherapy, and 13% received primary radiotherapy.^16^ Although our cohort included a relatively high proportion of FIGO (2009) stage III and IV tumors, primary radiotherapy was administered more frequently at each stage than previously reported.^16^, ^17^ Notably, nearly 75% of women with FIGO stage II disease received either adjuvant or primary radiotherapy to the vulva, reflecting the challenges associated with determining optimal treatment for larger perineal tumors without lymph node metastases. However, the limited sample size precludes definite conclusions and highlights the need for further investigation in prospective studies.

Although the surgical management of vulvar cancer has evolved toward less extensive surgery of the groins and vulva, complications remain high.^18^, ^19^ In our study, postoperative infections and wound dehiscence were more common than in a study evaluating tumors from all anatomical locations.^20^ The proximity of the anal region may contribute to an increased risk of infection and increased tissue tension, thereby impairing wound healing.

Only a small number of patients sought support from rehabilitation services, and fecal incontinence was infrequently documented in the medical records. This may reflect underreporting of treatment‐related side effects, as discussed by Hazewinkel et al. Patients may feel ashamed or perceive these symptoms as less important than the cancer diagnosis itself.^21^ Clinicians should therefore proactively inquire about pelvic floor disorders in vulvar cancer patients, particularly after treatment involving areas close to the anus.

Women treated with surgery only had low recurrence rates and excellent survival outcomes, consistent with previous studies of early‐stage disease.^22^, ^23^ The median surgical margin of 5 mm supports a more conservative, tissue‐sparing approach, which is particularly important in this anatomically sensitive location. Although the prognostic value of resection margins remains debated,^24^, ^25^ lymph node metastasis is a more robust predictor of outcome.^3^ Most women receiving adjuvant radiotherapy had lymph node metastases, and the vulva was frequently included in the radiation field. As lymph node, metastases are associated with a higher risk for local recurrence,^26^ ESGO guidelines recommend considering vulvar adjuvant radiotherapy in patients with groin metastases.^27^ However, a recent publication on the real‐world implementation of the ESGO 2023 guidelines highlighted substantial practice variation and persistent evidence gaps in adjuvant radiotherapy, underscoring the need for expert multidisciplinary consensus.^28^ Nevertheless, the high rates of skin toxicity observed in our study are noteworthy, particularly given the limited knowledge regarding complications associated with vulvar radiotherapy.^29^

The observed differences in relative and progression‐free survival between treatment groups were probably strongly influenced by baseline stage distribution and treatment indication. We observed a high rate of progression after primary radiotherapy, even after excluding patients with palliative intent. This likely reflects the preferential use of primary radiotherapy in patients with the most advanced disease, limiting direct comparisons with other treatment modalities. Another possible explanation is the occurrence of early inguinal recurrences due to insufficient primary treatment of groin metastases, as suggested by the GROINSS‐V II study.^30^ Conversely, after adjustment for stage, Bruce et al. suggested that primary radiotherapy may represent an acceptable alternative to surgery in women likely to require adjuvant radiotherapy.^31^

Given their anatomical proximity, the treatment of perineal vulvar squamous cell carcinoma may be aligned with that of anal cancer, in which primary radiotherapy plays a central role.^32^ However, anal cancer is more strongly associated with HPV than vulvar cancer.^33^ Notably, Hinten et al. reported a higher proportion of HPV‐associated tumors in the perineal region than in other parts of the vulva,^5^ and HPV‐positivity is a known predictor for favorable outcomes after radiotherapy in vulvar squamous cell carcinoma.^34^, ^35^ In our study, p16 analysis was available only for recent cases; nevertheless, resectable node‐negative disease appeared to have excellent outcomes despite largely unknown HPV status. In HPV‐independent tumors, more radical surgical treatment aimed at removing extensive vulvar dermatosis has been proposed to increase the likelihood of cure.^36^ In perineal vulvar squamous cell carcinoma, however, more extensive surgery may require resection of perianal or anal structures, resulting in substantial morbidity. Alternatively, neoadjuvant chemotherapy followed by wide local excision has been reported to be effective and well tolerated in younger women with sphincter‐threatening vulvar cancer.^37^ This strategy could potentially reduce the need for extensive surgery, adjuvant radiotherapy, or primary radiotherapy, although further investigation is required. The prospective, randomized controlled clinical VULCANize 2 trial is currently evaluating this treatment option.^38^

A strength of this study is the relatively large sample size of patients with vulvar squamous cell carcinoma located close to the anal region. Furthermore, the population‐based design, high data completeness and a long follow‐up period enabled reliable analyses of recurrence and survival outcomes. However, the study has several limitations, including its retrospective design, a heterogeneous study population, and small sample sizes within each treatment group, which precluded adjusted survival analyses. These are common challenges in studies of rare diseases. In addition, the imbalance in disease stage between treatment groups prevented causal comparisons of outcomes. Furthermore, data on HPV association were only available for more recent cases in the cohort. Another major limitation is the lack of patient‐reported outcomes, as complications were identified from medical records, which may have introduced information bias due to underreporting.

## 5 CONCLUSION

Primary surgery appears feasible for most patients with early‐stage vulvar squamous cell carcinoma located in the perineal region and is associated with excellent survival outcomes. In contrast, women with advanced‐stage disease had poorer outcomes and required surgery followed by adjuvant radiotherapy or primary radiotherapy. Owing to substantial differences in baseline disease characteristics, direct comparisons between treatment modalities were not possible. These findings highlight the importance of early diagnosis to achieve durable cure with limited morbidity. Further research is needed to establish the optimal treatment strategy for patients with advanced disease.

## AUTHOR CONTRIBUTIONS

Karin Kjölhede: Investigation, Conceptualization, Writing original draft. Gabriel Lindahl: Writing—reviewing and editing, Supervision. Diana Zach: Formal analysis, Writing—reviewing and editing, Supervision. Christian Staaf: Methodology, Writing—reviewing and editing. Katja Stenström Bohlin: Conceptualization, Methodology, Formal analysis, Writing—Reviewing and editing, Supervision, Funding acquisition.

## FUNDING INFORMATION

The study was supported by Anna‐Lisa och Bror Björnssons Foundation, Hjalmar Svensson Foundation, and National Highly Specialized Care funds (Swedish: NHV‐medel) of the Västra Götaland Region (NHV‐1012838).

## CONFLICT OF INTEREST STATEMENT

The authors declare having no conflict of interest associated with this manuscript.

## ETHICS STATEMENT

The study was approved by the Swedish Ethical Review Authority (Dnr 2020–00955, approved June 8, 2020; supplement Dnr 2021–06644‐02 approved January 11, 2022).

## Supporting information


**Figure S1.** Relative survival among women with treatment of perineal vulvar cancer excluding patients receiving palliative radiotherapy.
**Figure S2**. Progression‐free survival among women with treatment of perineal vulvar cancer excluding patients receiving palliative radiotherapy.

## Data Availability

The data that support the findings of this study are available on request from the corresponding author. The data are not publicly available due to privacy or ethical restrictions.
