# Supplementary material for: Treatment patterns and outcomes in perineal primary vulvar cancer—a population‐based Swedish cohort study
Source: Acta Obstet Gynecol Scand. 2026 Jul 22;105(11):2265–74. doi: 10.1111/aogs.70315 (PMC13394348; doi:10.1111/aogs.70315)
Supplement: Supplementary file 1 [file AOGS-105-2265-s001.docx]

Supplementary figures

Figure S1. Relative survival among women with treatment of perineal vulvar cancer excluding patients receiving palliative radiotherapy.

Figure S2. Progression free survival among women with treatment of perineal vulvar cancer excluding patients receiving palliative radiotherapy.
